# Using peer review to distribute group work marks equitably between medical students

**DOI:** 10.1186/s12909-017-0987-z

**Published:** 2017-09-20

**Authors:** Alex R Cook, Mikael Hartman, Nan Luo, Judy Sng, Ngan Phoon Fong, Wei Yen Lim, Mark I-Cheng Chen, Mee Lian Wong, Natarajan Rajaraman, Jeannette Jen-Mai Lee, Gerald Choon-Huat Koh

**Affiliations:** 1 Saw Swee Hock School of Public Health, National University of Singapore and National University Health System, Singapore, 117549 Singapore; 2 National University Hospital, Department of Surgery, National University Health System, Singapore, Singapore; 3 Communicable Disease Centre, Tan Tock Seng Hospital, Singapore, Singapore; 4 Yong Loo Lin School of Medicine, National University of Singapore and National University Health System, Singapore, Singapore

**Keywords:** Peer assessment, Group work mark, Individual contribution, Mathematical formulation

## Abstract

**Background:**

Although peer assessment has been used for evaluating performance of medical students and practicing doctors, it has not been studied as a method to distribute a common group work mark equitably to medical students working in large groups where tutors cannot observe all students constantly.

**Methods:**

The authors developed and evaluated a mathematical formulation whereby a common group mark could be distributed among group members using peer assessment of individual contributions to group work, maintaining inter-group variation in group work scores. This was motivated by community health projects undertaken by large groups of year four medical students at the National University of Singapore, and the new and old formulations are presented via application to 263 students in seven groups of 36 to 40 during the academic year 2012/2013.

**Results:**

This novel formulation produced a less clustered mark distribution that rewarded students who contributed more to their team. Although collusion among some members to form a voting alliance and ‘personal vendettas’ were potential problems, the former was not detected and the latter had little impact on the overall grade a student received when working in a large group. The majority of students thought the new formulation was fairer.

**Conclusions:**

The new formulation is easy to implement and arguably awards grades more equitably in modules where group work is a major component.

**Electronic supplementary material:**

The online version of this article (10.1186/s12909-017-0987-z) contains supplementary material, which is available to authorized users.

## Background

Peer assessment in medicine has been used to evaluate clinical performance of medical students and practicing doctors [1, 2]. Ramsey and colleagues have demonstrated that peer assessment is a reliable and valid method for assessing clinical performance in cognitive and psychosocial skills, both desired outcomes of medical education [3]. Others have found that trainer assessment by teaching staff is highly correlated with peer assessment by medical students [1, 4]. In programs where there are multiple opportunities for medical students to interact with and observe their peers, peer assessment of work habits is correlated with professional competencies such as problem identification and solving, independence, reasoning, and being well-prepared; and peer assessment of interpersonal habits was correlated with professional competencies such as understanding others, demonstrating respect, admitting mistakes, and trustworthiness [5, 6]. Although peer assessment is valued by medical students, further work is needed to understand how peer assessment can be used more effectively [7]. In particular, peer assessment has never been studied as a method to allocate a common group work mark equitably among medical students working on a joint project.

In group work, there are usually two methods to grade students within each group: students get either (i) the same mark as everybody else in the group because the ‘product’ assessed (e.g. a presentation or report) represents the whole group’s contribution, or (ii) an individual mark based on one’s contribution to the group work and ‘product’ assessed. The former has been criticized as unfair for it penalizes better students whose grade may be dragged down by weaker students, while giving weaker students a better grade than their contribution merits [8]. This approach also encourages free ridership as the penalty for one student not working hard is shared among the group, whereas the benefits to not working hard (for instance, focusing on other coursework) are reaped only by the student who “free-rides”. Although the second method of individual assessment is justifiably regarded as more equitable, it is not always possible for tutors to assign fair grades to all students on an individual basis: they may be unable to observe all members of the group working together for frequent long periods or are unable to monitor each member’s contributions outside direct observations. Instead, peers within the group are best placed to assess the contributions of each group member to a project and its ‘product’. Adding an individual peer review mark to an overall group mark is one obvious way to combine two outcomes, but this does not solve the free ridership problem and does not reward excellence as well as it should. A more equitable system that rewards excellence would give students who contributed more to the team’s work a greater share of the credit the team received. In this paper, we present a novel mathematical formulation to divide the credit encapsulated in a single group work mark between members of a group using peer-assessed contribution of each member to their group’s work.

## Methods

### Group work module

In the Yong Loo Lin School of Medicine, National University of Singapore, medical students in the fourth year of a 5 year bachelor of medicine and bachelor of surgery degree embark on a research project over a year in a module called the Community Health Project (CHP), supervised by faculty members from the Saw Swee Hock School of Public Health in the same university. The CHP is a capstone research project in which groups of medical students (of 25–40 students each) work on a clinical, public health, epidemiologic or health services research project with the ultimate goal of better understanding and improving the health status of their target population, and which is often published (for examples see Wee et al. [9], Foo et al. [10], Lee et al. [11] or Koo et al. [12]). Medical students are expected to integrate and apply what they have learned from basic sciences, clinical sciences, epidemiology, public health and biostatistics since the start of medical school in their CHP. The research population may be people from the community, underserved populations (e.g. migrants, low income and elderly) or institutions (e.g. hospitals, specialist outpatient centers and primary healthcare clinics). Groups typically divide themselves into teams responsible for one aspect of the overall endeavor—for instance, teams for literature review, report writing, data analysis, presentation, institutional review board application, or study coordination—with all students typically involved in data collection (usually surveys conducted on 500 to 1000 individuals) and with most of the project taking place over an intensive 6 week period in which they take no other classes. Groups present their research findings at a school-wide conference attended by fellow students and academics, and each group submits a report. The quality of these presentations and reports—the ‘product’ of the CHP group work—are assessed by a panel of faculty members. Heretofore, each student shared the same common presentation and report mark for the group. In addition, every student also assessed the quality and quantity of contributions by each member in their own group, and the peer assessment scores were added to the group mark to give their final CHP mark. These peer assessment scores were unique for each student and represented their individual contribution to their CHP and their ‘individual’ mark. However, there was dissatisfaction among the faculty and student body that, with every member being awarded the same score for their group’s presentation and report, weaker students were being unfairly rewarded and harder working students under-recognized. Moreover, as there may be inter-group variation (one group may be more or less generous in peer assessment than other groups), the mean peer review scores of one group could be significantly different than in another group. Hence, we sought to develop an alternative mathematical formulation that achieves two goals: (i) the group mark (for the presentation and report) is more equitably distributed between the members of the group to reflect their contributions to the research project, and (ii) to control for inter-group variation in peer assessment scores (by standardizing the peer assessment scores of each member within each group to the mean score of the group) while maintaining inter-group variation in group work scores. This new mathematical formulation was first implemented in the 2012–2013 academic year and has been used in all academic years since.

### Student peer assessment guidelines

At the start of the year-long CHP, medical students were informed that (i) the module was a collaborative group project that demanded contributions from all group members, (ii) active participation was a requirement and their individual contributions would be factored into allocation of the common group mark for their group’s presentation and report towards their individual mark, and (iii) they would be asked to evaluate the participation of their classmates in a fair and honest manner, based on observation of their peers’ participation and contributions in class, field work and online discussions at the end of year/project. We gave them positive examples of peer participation and contributions (as suggested by Didicher [13]) which are detailed in Table 1.

**Table 1:** Positive examples of group participation and contributions

Arriving punctually at meetings	
Contributing to class discussions, including both questioning and answering	
Using positive listening skills (e.g. paying attention when a colleague is speaking, being interested, positive body language, and sharing one’s own perspective in a productive and supportive manner)	
Responding to other students during discussion	
Being prepared to discuss questions, problems and other issues in class	
Active behaviors (e.g. collecting or analyzing data, writing and presenting)	
Summarizing group notes recorded during class and posting them online in collaborative project management platforms (e.g. Yahoo! Groups, Google Docs and Dropbox)	
Managing group work (e.g. being a time-keeper, keeping people on track for tasks and organizing people)	
Giving feedback to fellow students on their work, either in class or online	
Posting on online discussion boards (e.g. Facebook and Wikispaces), beginning or adding to discussions	
Collaboration in class, outside class and in online activities	
Sharing learning and research resources with fellow students	
Teaching fellow students research skills	

At the end of their CHP, students were asked to consider the quantity (e.g. attendance and time spent on the aforementioned examples of participation) and the quality (e.g. competency, responsibility, initiative, cooperation, clarity, creativity and enthusiasm) of contributions, and score the overall contribution of each of their fellow group member on a 6-point Likert scale: 1 = very below average, 2 = below average, 3 = low average, 4 = high average, 5 = above average, 6 = very above average. Because of the extremely varied nature of students’ possible contributions to the group, it was not feasible to be more explicit in what behaviors were good examples of each point on the Likert scale. Consistency of ratings was assessed using a modified form of Cronbach’s α statistic, adjusted to account for the systematic missingness due to students’ not assessing themselves. Specifically, if *X*_*ij*_ is the mark *i* gave *j*, for *i*, *j* ∈ {1, …, *n*} in the same group, and ${T}_j=\sum_{i\ne j}{X}_{ij}$, ${\sigma}_{X^2}=V\left({T}_j\right)$ is the variance over values of *j*, and *σ*_*Yj*_ = *V*(*X*_*ij*_) over values of *i*, then we let

$$ \alpha =\left(1-\frac{\sum_j{\sigma}_{Yj}}{\sigma_{X^2}}\right)\frac{n}{n-1}. $$

### Mathematical method

We denote the group to which individual *i* belongs by *G*_*i*_. Let the score for the group work component be *S*_*G*_ for group *G* (in our case, this is the average of the faculty’s evaluation of the group’s submitted report and presentation, and is out of 100). Let *p*_*ji*_ be the peer evaluation score (ranging from 1 to 6) which a peer, *j*, gives individual *i*. We have used two ways to create an overall peer evaluation score for individual *i*.

The first is a straightforward average of the other students’ evaluations, given by:

$$ {P}_i^1=\sum_{j\ne i}{p}_{ji}/\left({n}_{G_i}-1\right) $$

where ${n}_{G_i}$ is the number of students in group *G*_*i*_ and the summation is over all other peers *j* in the same group as *i*. For our module, typically the number of students per group is around 25 to 40, and there are around 6 to 8 groups per academic year. Although this formulation has the benefit of clarity, it is at the expense of equity for a student can give all peers a high score without ill consequence.

The second way is to standardize relative to each assessor’s average. This is equivalent to giving each student a number of points to distribute among peers proportional to their perceived effort. If we denote

$$ {g}_j=\sum_{k\ne j}{p}_{jk}/\left({n}_{G_j}-1\right) $$

to be the ‘generosity’ (*g*_*j*_) of student *j* (i.e. the average points *j* awards her peers), where again a dummy variable *k* stands for others in the group, then the score *j* awards *i* relative to *j*’s average is

$$ {p}_{ji}^{\prime }=\frac{p_{ji}}{g_j}. $$

Thus to derive *i*’s score under this scheme, we simply take the average of the scaled points given him by each student, *j*:

$$ {P}_i^2=\sum_{j\ne i}{p}_{ji}^{\prime }/\left({n}_{G_i}-1\right). $$

For large groups, ${P}_i^2\approx {P}_i^1$, as the effect of each student’s differing standards is minimal, but the use of the second is fairer within smaller groups and ensures consistency between groups. Both can readily be calculated using a spreadsheet such as Microsoft Excel or statistics software such as R [14]. The Additional file 1 contains a worked example using Microsoft Excel which can be adapted by future adopters.

Our old scoring system took a straight-forward weighted average of the group component, shared by all members of the group, and peer review, to give the student’s overall score for the module as:

$$ {F}_i^{old}=\frac{w_1\times {S}_{G_i}+{w}_2\times \left(100\times {P}_i/6\right)}{w_1+{w}_2} $$

using either peer evaluation formulae ${P}_i^1$ or, preferably, ${P}_i^2$, and with peer review score rescaled to 0–100 to be on a consistent scale with the shared group component. Traditionally we assigned weights *w*_1_ = 4 to the group component and *w*_2_ = 3 to the peer review.

The new system distributes the group score of the report and presentation by the contribution of each team member as determined by the peer evaluation score, thus incentivizing individual contribution to the team’s effort to raise both the team’s score and one’s own share of it. The formula we developed was ${F}_i^{new}={S}_{G_i}\times {P}_i$, i.e. the group score multiplied by the peer review score (again, either peer evaluation formula ${P}_i^1$ or ${P}_i^2$ could be used, though the latter is preferable). A complication in the new system is that occasionally a student may obtain a score greater than 100. The simplest solution is just to award this student the maximum mark (i.e. 100). A more elegant alternative would be to award that student 100 marks and redistribute some of the surplus to the other members of that individual’s team, an alternative that is substantially more complicated to implement.

To illustrate the difference between the old and new system, we compared ${F}_i^{old}$ with ${F}_i^{new}$ using peer evaluation formula ${P}_i^2$ on the presentation, report and peer assessment scores from a cohort of students who underwent the CHP module from May 2012 to March 2013. Within this cohort, there were seven groups (labeled A to G) with 36 to 40 students per group. Results for all groups are presented in the results section, obtained using a series of scripts in the R statistical environment [14], while a worked example using Microsoft Excel can be found as Supplementary Information for one group. Graphs were created using scripts that made heavy use of the grid package in R [15]. We also surveyed a subsequent cohort of students at the completion of their CHP (in 2014) on their perceptions of the fairness of the new and old systems (feedback form provided in Additional file 3).

### Simulation study

To assess the impact of a student’s effort and ability on her assessed performance using peer review, we conducted a small simulation study. In this, we simulated 300 students in ten groups of *n* = 30 (results were similar for smaller groups and are not shown) and randomly assigned each student an *ability* parameter, *A*_*i*_ ~ *N*(100, 25) and an *effort* parameter *E*_*i*_ ∼ *Be*(0.5, 0.1) or *Be*(0.75, 0.1), both distributions parameterized by the mean and standard deviation. The *contribution* of student *i* was defined to be *C*_*i*_ = *A*_*i*_ *E*_*i*_. The group score was set to be the average of all students’ contributions within the group, i.e. the group score for group *g* was ${S}_g=\sum_{i:{G}_i=g}{C}_i/n$ where *G*_*i*_ is the group *i* belongs to. We then systematically varied one focal individual’s ability and effort.

We assumed three different processes for the peer review. In one, each student was assessed based on his ability, but not on his effort. In the second, he was assessed based on his effort only. In the third, he was assessed on his contribution, i.e. both. We then calculated the score obtained using the new system and without using peer review, and calculated his rank in the class. This was repeated 100 times for each combination of effort, ability, and the factor being assessed by his peers (effort, ability or both).

## Results

**Fig. 1:**
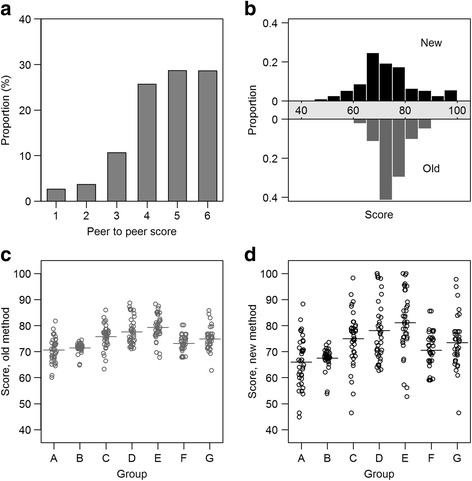
Peer to peer score distribution, and new and old community health project marking systems. **a** Distribution of scores on 6-point Likert scale given by students to their peers before aggregation. **b** Comparison of the overall distribution of marks between the two systems. **c** Using the old system. Each circle represents one student; the x-axis is jittered for visual acuity. Horizontal lines indicate group averages. **d** Using the new system

The raw scores given by our students to their peers were typically above ‘average’ (Fig. 1(a)) with mean 4.6 out of 6 and standard deviation 1.3. After implementing the new scoring system, although the mean student scores for all seven groups remain largely unchanged (overall, from 71 to 73 points on a 100-point scale), the dispersion of final marks widened considerably from the old method [Fig. 1(c), overall standard deviation, *s* = 4 points] to the new [Fig. 1(d), *s* = 11 points]. Thus, one effect of switching from the old to the new system was making the students’ marks more heteroskedastic or “spread out” (i.e. making it easier to reward excellence and detect underperformers) [Fig. 1(b)].

**Fig. 2:**
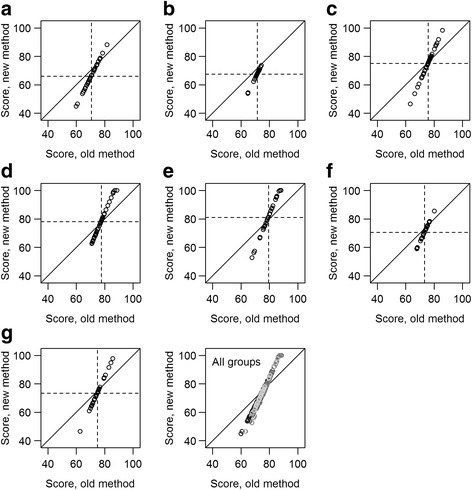
Individual student scores within groups (**a**–**g**) and overall under old and new methods. Membership of teams in the *all groups* panel is indicated by shade of gray. Dashed lines indicate mean within groups, and the solid line is the line of equality. Ordering of students within groups was preserved, while ordering within the class was quite closely preserved (panel h, score correlation r = 0.86, *p* < 0.001) when moving to the new system, although individuals who contributed more to their groups, as measured by their peers, benefitted under the new system, while those who did not contribute much to their team were penalized

While the students’ marks were more spread out, the ordering of the students by marks was preserved within groups [Fig. 2] and fairly closely preserved in the class as a whole in the new system [Fig. 2, *r* = 0.86, *p* < 0.001], though students who contributed substantially more, or less, than average saw their ranks change accordingly. The biggest effects were on students with lower peer review scores in groups with higher group assessment scores, and those with higher peer review scores in groups with lower group assessment scores.

**Fig. 3:**
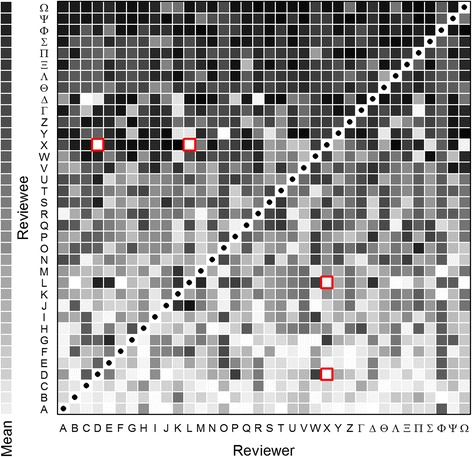
Individual peer review scores for one group (*n* = 36, group G in the other figures). Reviewers and reviewees are indicated by (the same) Roman and Greek capital letters. Normalized z scores were converted to grey-scale with highest scores in black and lowest scores in white (so, for example, student Ω received many high scores while student A received few). Black circles on a white background indicate missing values as individuals did not score themselves. Average marks on the same scale are presented on the strip to the left of the box for the same 36 students. Red boxes highlight two pairs of students (X-D and X-L) who seem to have given each other lower scores than they ought to, based on how their peers scored them

To assess whether a clique had formed a voting alliance, we created visual representations of voting patterns within groups to check for obvious collusion [see Fig. 3 and Additional file 2: Figure S1 for examples of one group] but none was apparent. Some student pairs (e.g. X-D, X-L, highlighted in red in Fig. 3 and very obvious in the Additional file 2: Figure S1) appeared to award each other lower marks than expected based on the rest of the group, but the effect of this was minimal on the final peer review scores, so no correction was attempted. Cronbach’s α statistics were high (across groups, the median being 0.88, and the inter-quartile range being 0.82 to 0.93) indicating the good internal consistency of the peer review process.

Out of 253 participants in the 2013–14 CHP, *n* = 249 (98%) took part in a module evaluation which included questions on their perceptions of the fairness of (*n* = 248) and preferences for (*n* = 247) the grading schemes. The majority reported finding the new system fairer (85%, 95% confidence interval [CI] 82–91%) and preferred it to the old system (79%, 95% CI 75–86%).

**Fig. 4:**
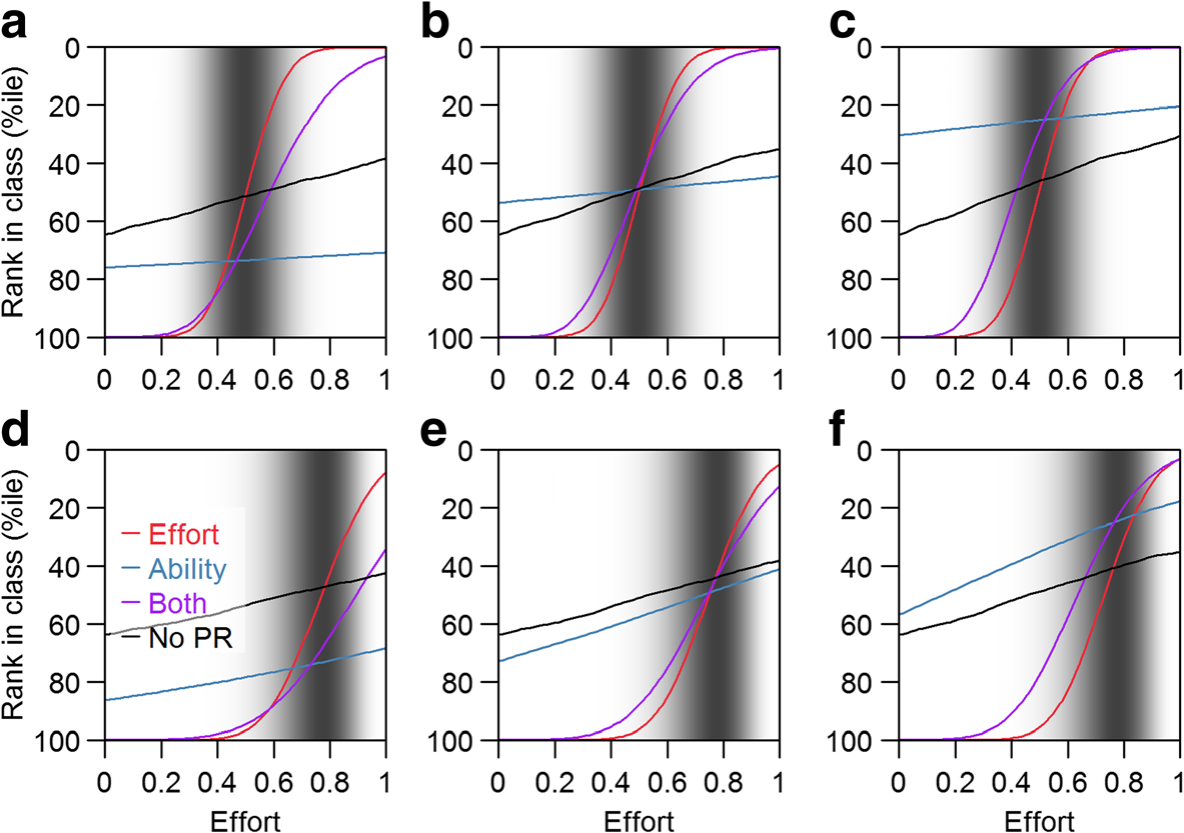
Simulation study effects of varying efforts, ability and what peers use to assess each other on ranking in class. The gray shading indicates the distribution of effort put in by others in the class (on an arbitrary scale); these distributions differ for panels (**a**) to (**c**) and for (**d**) to (**f**). The x-axes indicate the amount of effort put in by one focal student, on the same scale. Panels (**a**) and (**d**) are for a student in the 25%ile of ability, (**b**) and (**e**) for the median, and (**c**) and (**f**) for the 75%ile of ability. The colored lines signify scenarios in which team mates assess each other based on the effort they put in (red), their ability (blue) or their contribution (the product of the two, purple). In black is represented the rank if no peer review is used and the students are graded solely based on their team’s product

The simulation study presented in Fig. 4 shows the potential motivating effect of the scoring system. For all levels of ability, as long as students’ peers assess them based on either their effort (red lines) or their contributions (purple lines, which we quantify as the product of their effort and their ability) to the group work, it is possible to increase their ranking in the class by expending more effort on the group work or, contrariwise, for even a strong student to receive a low score if their contributions are minimal. This is in contrast to the situation where there is no peer review component (black lines), where the same increase in individual effort results in a much more modest increase in the student’s placing in the class as a whole, as the rewards of that increase in effort are shared with the rest of the team, and a reasonable grade can be achieved with minimal effort as long as others in the team contribute. Peer review becomes ineffective in this regard, however, if the assessment is based on the student’s ability rather than his or her efforts (blue line).

Following this logic, students are incentivized to increase their contributions to the group, but so are their team mates, which would lead to a shift in the distribution of effort (from Fig. 4a–c to Fig. 4d–f) that means even greater effort is needed to score higher.

## Discussion

A fundamental limitation of our study is that our group sizes were larger than in most team-based education where teams of <10 students are the norm, and more testing of our approach in smaller groups is needed before we can safely recommend it in those scenarios. In smaller groups, we anticipate that care should be taken to ensure both anonymity and the perception of anonymity, and instructors may wish to approve peer review scores. We expect that, under the new system, the smaller teams are, the greater will be students’ incentives to contribute, as their increased participation would result in a more direct impact on the group score and thus better rewarding every extra contribution. Smaller groups would also, we believe, improve the reliability of the peer review process itself, with students better able to assess all group mates’ contributions, and would allow the measurement tool to be extended from a single Likert scale to assess multiple dimensions of each team mate’s contribution. However, careful attention would be required to ensure that no harm is introduced by incorporating peer review into the assessment in smaller groups, and research to determine the validity of the peer review in small groups would be valuable.

Those limitations notwithstanding, in our large group setting, the overall effect of switching to the new system was to raise the group work mark of students who participated more and to lower the mark of those who contributed less, while controlling for inter-group variation in peer assessment scores but retaining inter-group variation in group work scores. In the absence of peer review, there is the tragedy of the commons [16], because each student received only a small portion of the benefits resulting from each extra unit of effort invested, the rest going to the student’s team mates, regardless of their contribution. The new system incentivizes each extra unit of effort, as the greater the student’s contribution, the greater the fraction of the resulting rewards that student receives; it also strongly disincentivizes free ridership, for a student perceived to contribute nothing to the team receives very little of the rewards of the team’s efforts (see the outlying students in Fig. 2). Allowing those who contribute a lot to increase their share of the marks, while preventing those who contribute little from benefiting from their peers’ work, leads to greater spread in the marks at the class level, a reflection of the greater accountability under the new system. The multiplicative system means that a student of modest ability in a weak group might receive an outstanding peer review score, but if the group did not achieve much, that student will receive a high proportion of a small group score, which may be more commensurate with the student’s contributions than if an additive system were used.

As far as we are aware, this is the first paper to describe the use of peer assessment to evaluate individual contribution to group project work with an (arguably) equitable formulation that uses peer-assessment scores to allocate individual marks from a common group work mark. Standardizing peer review scales circumvented any issues of ‘grade inflation’ by peer reviewers and the problem of differing standards, as it has been observed that low achieving students tend to score their peers more generously by averaging out these variations within the group [17]. As peer assessment of colleagues’ contribution to group work includes peer assessment of work and inter-personal habits, it is likely that the final scores are correlated with professional competencies as described by Dannefer et al. [5]. The new system also overcomes the injustice of better students being ‘dragged down’ by weaker students in the group, and of lazier students having a ‘free ride’ on the work of more motivated students, otherwise associated with a common group work mark. This relies on peer-observed assessment of individual contribution, which we believe is more accurate than teacher-observed assessment for large classes, and in our class was found to have high internal consistency. However, as the simulation study we presented shows, the impact of peer review in determining a student’s final grade depends strongly on what group mates assess: if it is perceived that they assess ‘ability’ rather than effort, we would expect much less incentivization to contribute to the group, and the peer review system would be no better in encouraging effective group work than having no peer review component at all. We believe that the positive examples of group participation that we provide students (Table 1) may frame expectations of how peer review should be conducted to effect desired outcomes.

A downside to the increased importance of peer evaluation scores is the risk that students may try to game the system (e.g. by colluding to form a voting alliance to improve their grades). However, we did not find evidence of this. Another, realized problem is that some pairs of students may award each other unusually low grades [for instance, students pairs “X and D” and “X and L” in Fig. 3] for ostensibly personal reasons. While such ‘personal vendettas’ had very little impact on the overall grade a student received in our large groups, checking for this may be important in smaller groups. For classes in which the project work is very structured, it may be possible, and would be desirable, to provide concrete behavioral anchoring in the peer review process.

The panel assessing the group reports and presentations, who were not involved in the supervision, discerned a higher quality in the outcomes assessed compared to groups from previous years under the old system. Future work systematically quantifying changes in motivation would be valuable.

## Conclusions

The use of peer-assessed contributions to allocate individual marks from a common group work mark via a novel mathematical formulation produced an arguably fairer and less-clustered distribution, adjusted for inter-group variation in peer assessment while retaining inter-group variation in group work scores, compared to merely summing common group work and individual peer assessment scores. Although collusion among some members to form a voting alliance and ‘personal vendettas’ were potential problems, we could not detect the former and the latter had little impact on the overall grade a student received when working in a large group. The new system rewards students who contribute more, and penalizes those who contribute less, incentivizing desirable behavior.

## Additional files

Additional file 1: Worked Example. Worked example in excel for the group featured in **Fig. 3** The *rawdata* tab contains the raw score (out of six) awarded by each individual to each other individual (individuals’ names are replaced by majuscule Roman or Greek letters) in blue tinted cells. These are converted to a modified score (orange tinted cells) by scaling by the donor’s overall mean donated score (his or her ‘generosity’). The *processed* tab converts these to a final score out of 100 for each student. (XLSX 43 kb)

Additional file 2: Figure S1. Each page represents one student, whose names have been replaced by Roman or Greek capitals. The index student referred in each page is indicated by the down arrow (↓) on the top row of letters. The upper chart indicates the marks the index student gave to each peer (black or colored circles), together with the average points awarded to that peer (grey bars). The lower chart indicates the marks each peer gives the index student (circles) and the average mark the index student received. Note: these marks are the raw marks prior to scaling. If any mark is more than 1.5 marks away from average, this is indicated by coloring the circle (orange for less, red for much [2.5] less, light blue for more, dark blue for much [2.5] more), increasing the shading on the bar, and adding an arrow. By maximizing the graph on screen and running through each page as a slide show, faculty can quickly assess for the presence of collusion between students. (PDF 140 kb)

Additional file 3: CHP 2014 Module Feedback Form. Form used to solicit feedback on the scoring system. (PDF 13 kb)

## Abbreviation

CHP: Community Health Project
