# Supplementary material for: Using peer review to distribute group work marks equitably between medical students
Source: BMC Med Educ. 2017 Sep 20;17:172. doi: 10.1186/s12909-017-0987-z (PMC5607620; doi:10.1186/s12909-017-0987-z)
Supplement: Supplementary file 2 — Each page represents one student, whose names have been replaced by Roman or Greek capitals. The index student referred in each page is indicated by the down arrow (↓) on the top row of letters. The upper chart indicates the marks the index student gave to each peer (black or colored circles), together with the average points awarded to that peer (grey bars). The lower chart indicates the marks each peer gives the index student (circles) and the average mark the index student received. Note: these marks are the raw marks prior to scaling. If any mark is more than 1.5 marks away from average, this is indicated by coloring the circle (orange for less, red for much [2.5] less, light blue for more, dark blue for much [2.5] more), increasing the shading on the bar, and adding an arrow. By maximizing the graph on screen and running through each page as a slide show, faculty can quickly assess for the presence of collusion between students. (PDF 140 kb) [file 12909_2017_987_MOESM2_ESM.pdf]

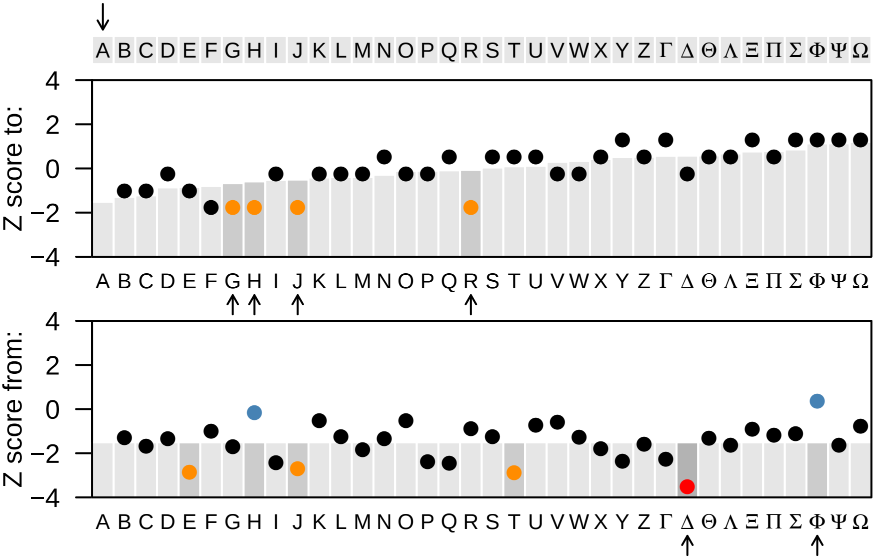
A B C D E F G H I J K L M N O P Q R S T U V W X Y Z Γ Δ Θ Λ Ξ Π Σ Φ Ψ Ω
Z score to:
4
2
0
−2
−4
A B C D E F G H I J K L M N O P Q R S T U V W X Y Z Γ Δ Θ Λ Ξ Π Σ Φ Ψ Ω
Z score from:
4
2
0
−2
−4
A B C D E F G H I J K L M N O P Q R S T U V W X Y Z Γ Δ Θ Λ Ξ Π Σ Φ Ψ Ω

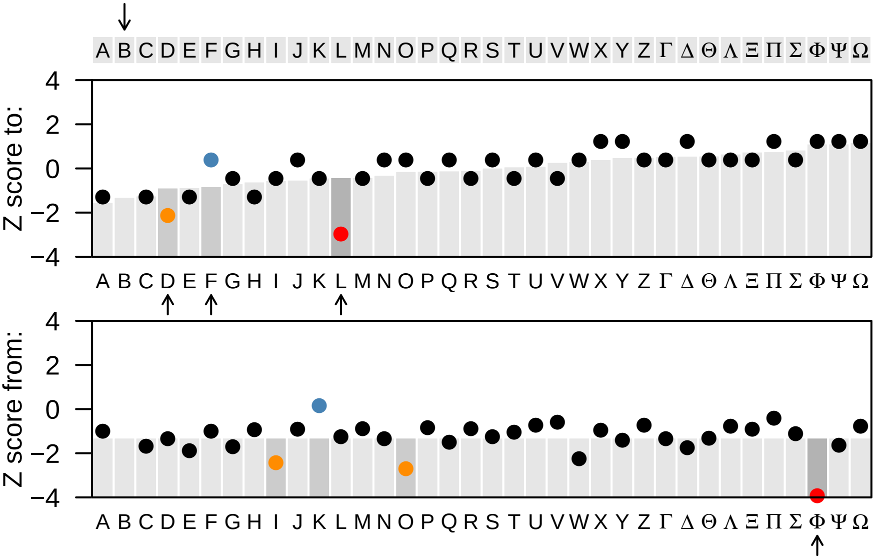
A B C D E F G H I J K L M N O P Q R S T U V W X Y Z Γ Δ Θ Λ Ξ Π Σ Φ Ψ Ω
Z score to:
4
2
0
−2
−4
A B C D E F G H I J K L M N O P Q R S T U V W X Y Z Γ Δ Θ Λ Ξ Π Σ Φ Ψ Ω
Z score from:
4
2
0
−2
−4
A B C D E F G H I J K L M N O P Q R S T U V W X Y Z Γ Δ Θ Λ Ξ Π Σ Φ Ψ Ω

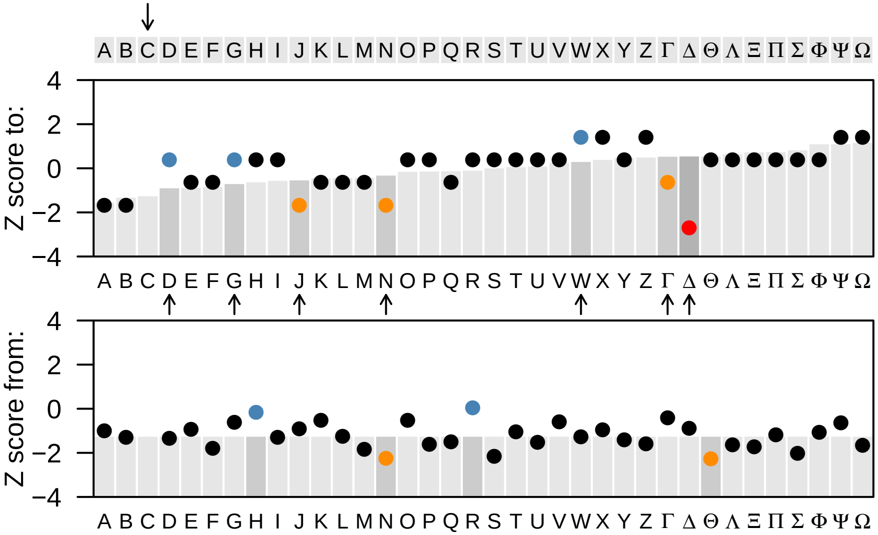
A B C D E F G H I J K L M N O P Q R S T U V W X Y Z Γ Δ Θ Λ Ξ Π Σ Φ Ψ Ω
Z score to:
4
2
0
−2
−4
Z score from:

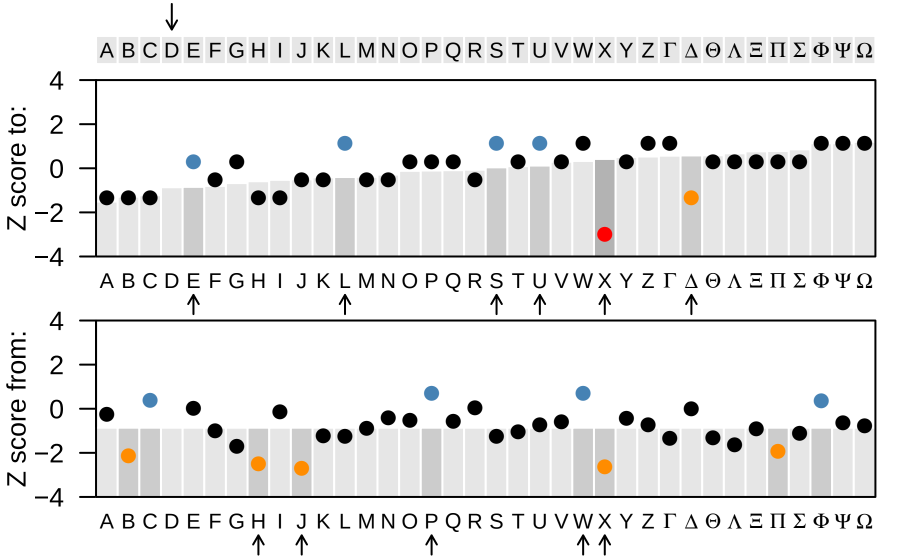

A B C D E F G H I J K L M N O P Q R S T U V W X Y Z Γ Δ Θ Λ Ξ Π Σ Φ Ψ Ω
Z score to:
Z score from:
4
2
0
−2
−4

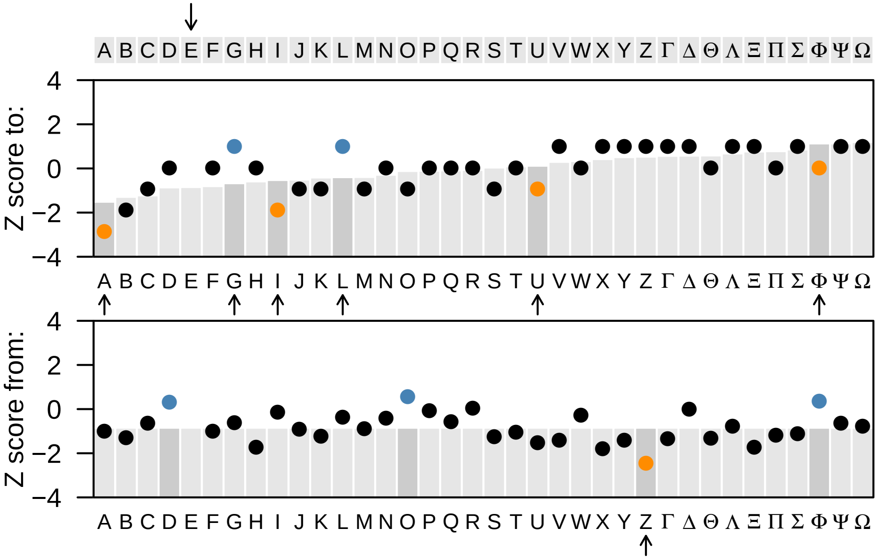

A B C D E F G H I J K L M N O P Q R S T U V W X Y Z Γ Δ Θ Λ Ξ Π Σ Φ Ψ Ω
Z score to:
Z score from:
4
2
0
−2
−4

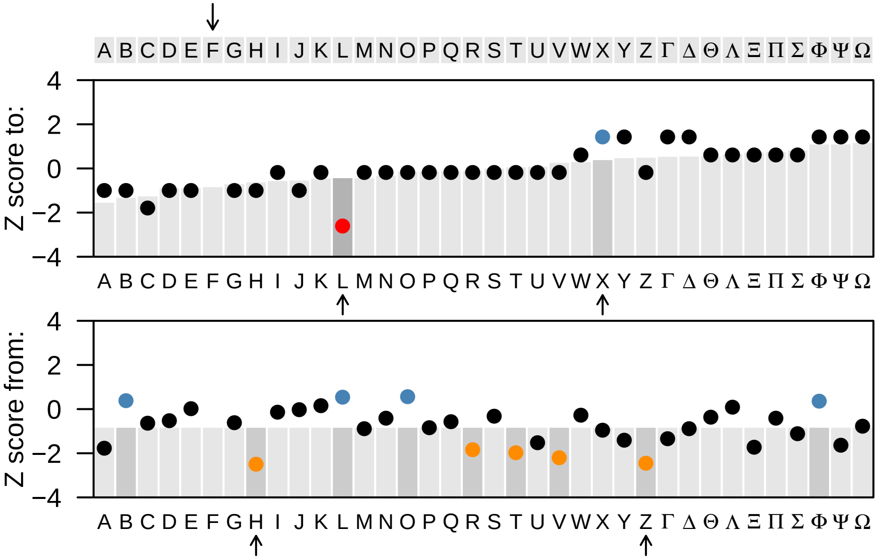
A B C D E F G H I J K L M N O P Q R S T U V W X Y Z Γ Δ Θ Λ Ξ Π Σ Φ Ψ Ω
Z score to:
4
2
0
−2
−4
Z score from:
4
2
0
−2
−4

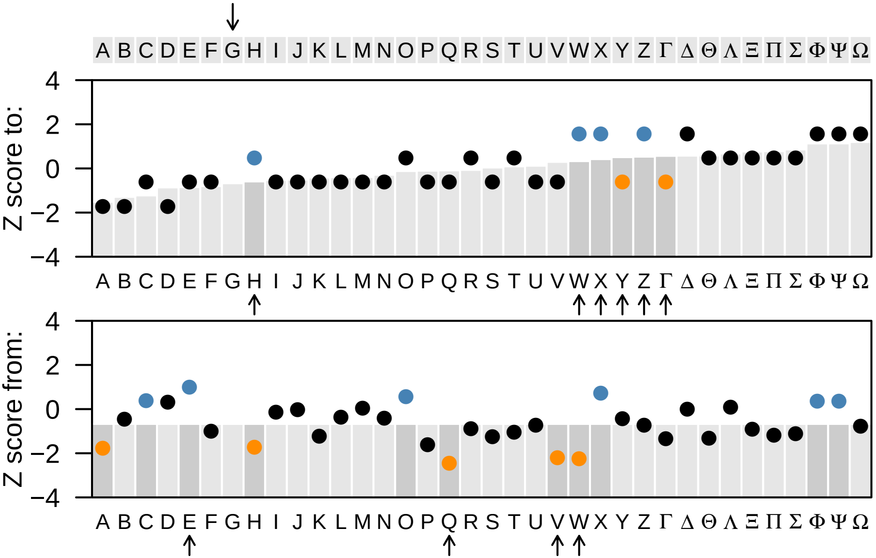

A B C D E F G H I J K L M N O P Q R S T U V W X Y Z Γ Δ Θ Λ Ξ Π Σ Φ Ψ Ω
Z score to:
4
2
0
−2
−4
A B C D E F G H I J K L M N O P Q R S T U V W X Y Z Γ Δ Θ Λ Ξ Π Σ Φ Ψ Ω
Z score from:
4
2
0
−2
−4
A B C D E F G H I J K L M N O P Q R S T U V W X Y Z Γ Δ Θ Λ Ξ Π Σ Φ Ψ Ω

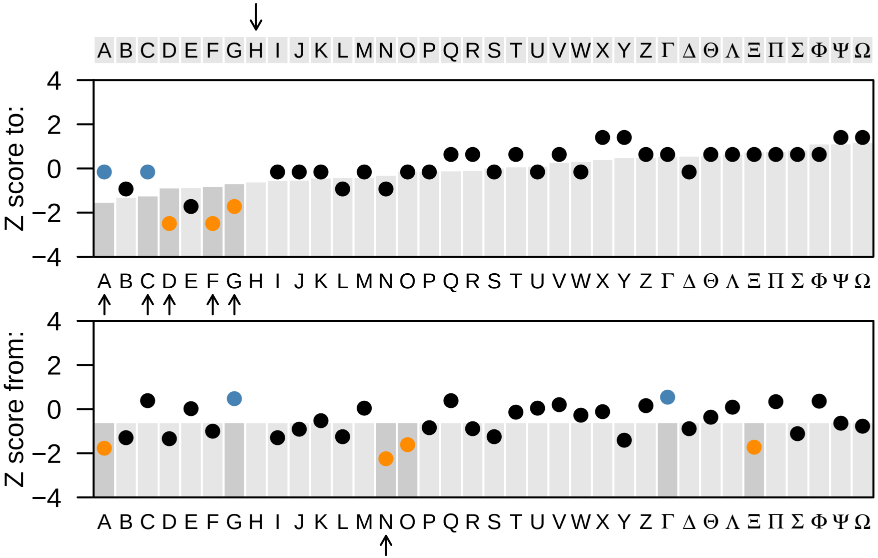

A B C D E F G H I J K L M N O P Q R S T U V W X Y Z Γ Δ Θ Λ Ξ Π Σ Φ Ψ Ω
Z score to:
4
2
0
−2
−4
Z score from:

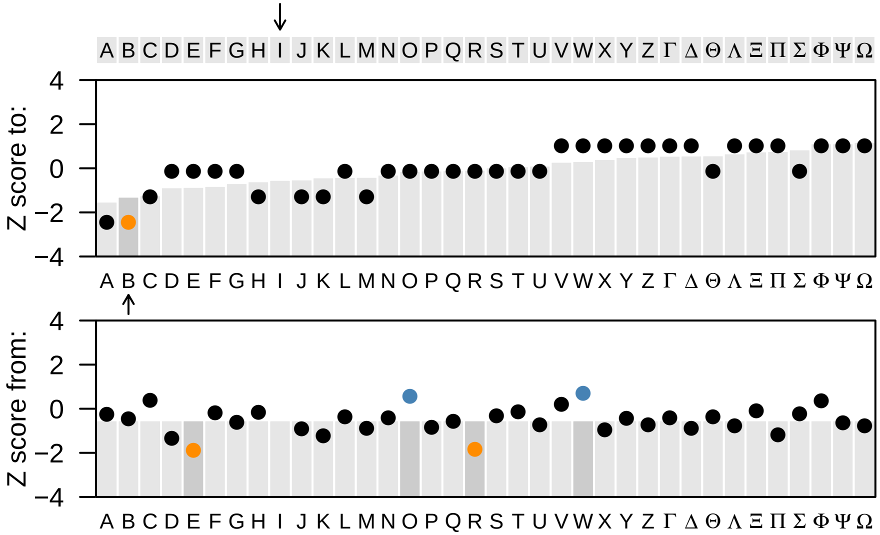

A B C D E F G H I J K L M N O P Q R S T U V W X Y Z Γ Δ Θ Λ Ξ Π Σ Φ Ψ Ω
Z score to:
4
2
0
−2
−4
A B C D E F G H I J K L M N O P Q R S T U V W X Y Z Γ Δ Θ Λ Ξ Π Σ Φ Ψ Ω
Z score from:
4
2
0
−2
−4
A B C D E F G H I J K L M N O P Q R S T U V W X Y Z Γ Δ Θ Λ Ξ Π Σ Φ Ψ Ω

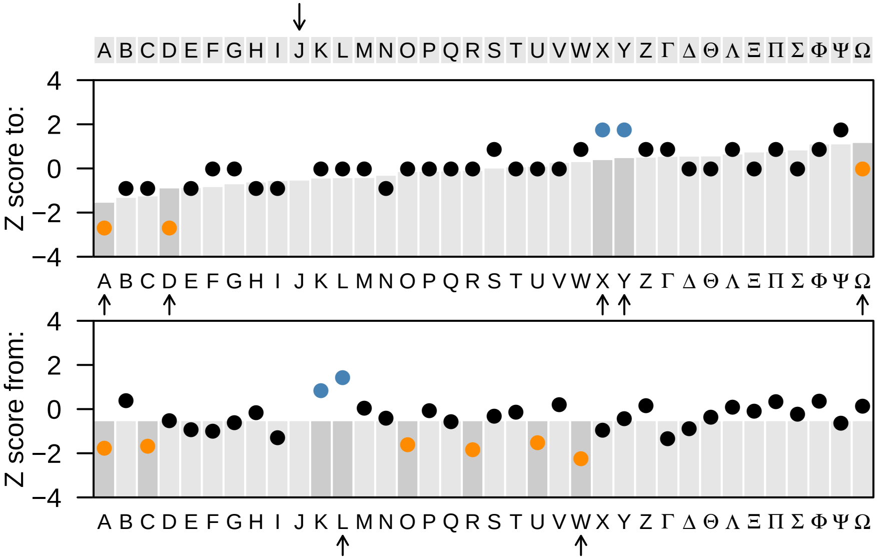
A B C D E F G H I J K L M N O P Q R S T U V W X Y Z Γ Δ Θ Λ Ξ Π Σ Φ Ψ Ω
Z score to:
Z score from:
4
2
0
−2
−4

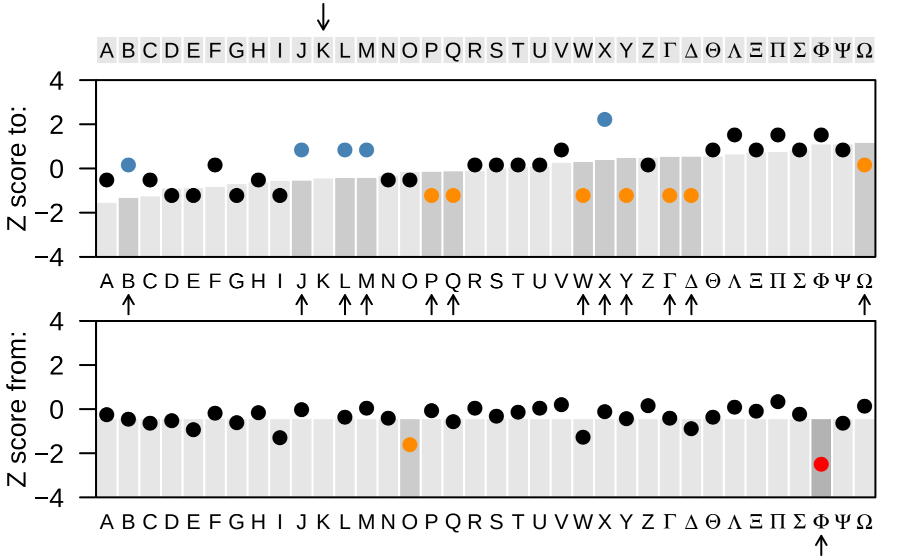

A B C D E F G H I J K L M N O P Q R S T U V W X Y Z Γ Δ Θ Λ Ξ Π Σ Φ Ψ Ω
Z score to:
Z score from:
4
2
0
−2
−4

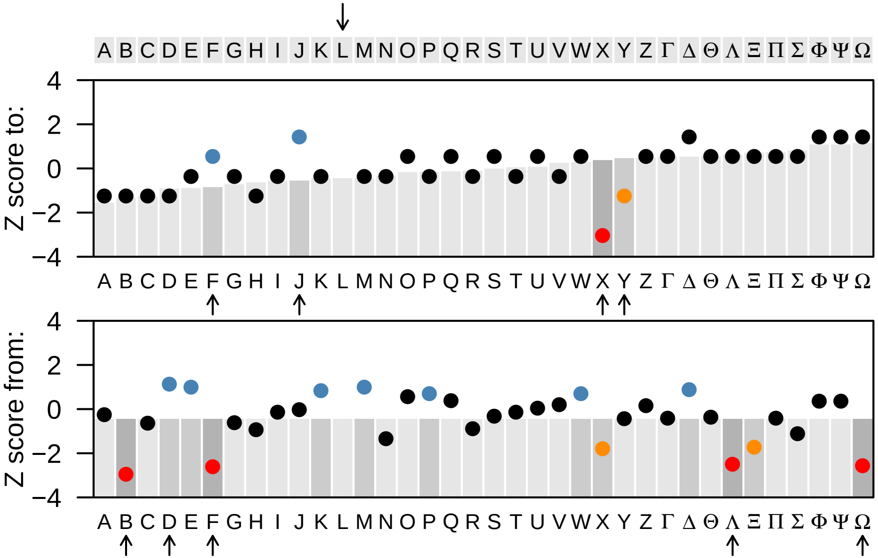

A B C D E F G H I J K L M N O P Q R S T U V W X Y Z Γ Δ Θ Λ Ξ Π Σ Φ Ψ Ω
Z score to:
Z score from:
4
2
0
−2
−4

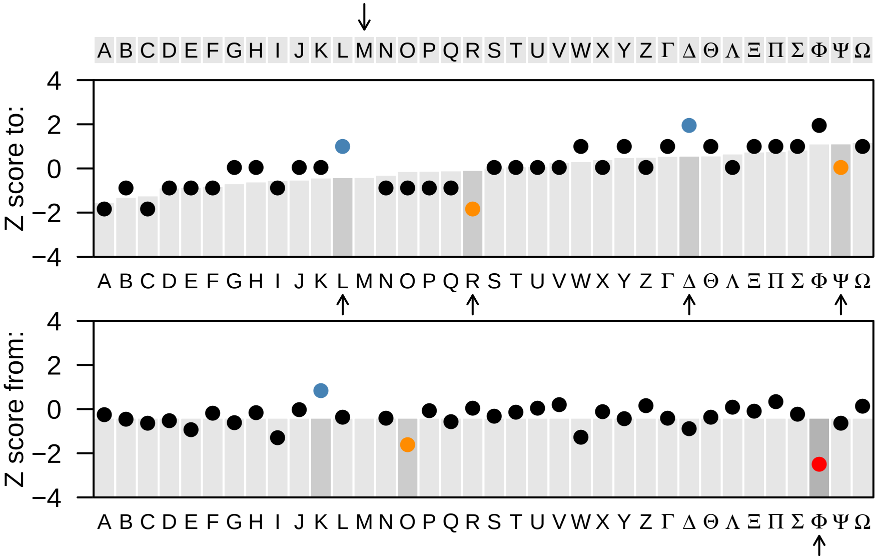

A B C D E F G H I J K L M N O P Q R S T U V W X Y Z Γ Δ Θ Λ Ξ Π Σ Φ Ψ Ω
Z score to:
4
2
0
−2
−4
A B C D E F G H I J K L M N O P Q R S T U V W X Y Z Γ Δ Θ Λ Ξ Π Σ Φ Ψ Ω
Z score from:
4
2
0
−2
−4
A B C D E F G H I J K L M N O P Q R S T U V W X Y Z Γ Δ Θ Λ Ξ Π Σ Φ Ψ Ω

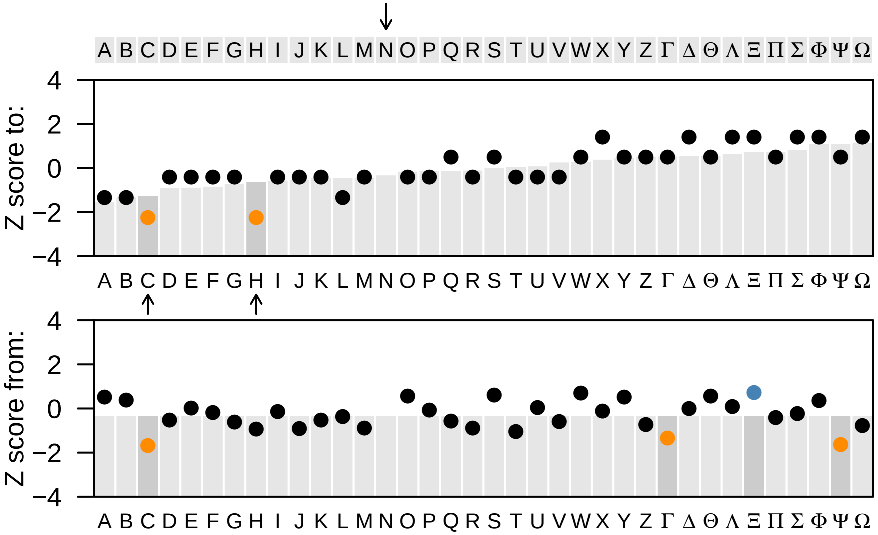

A B C D E F G H I J K L M N O P Q R S T U V W X Y Z Γ Δ Θ Λ Ξ Π Σ Φ Ψ Ω
Z score to:
4
2
0
−2
−4
A B C D E F G H I J K L M N O P Q R S T U V W X Y Z Γ Δ Θ Λ Ξ Π Σ Φ Ψ Ω
Z score from:
4
2
0
−2
−4
A B C D E F G H I J K L M N O P Q R S T U V W X Y Z Γ Δ Θ Λ Ξ Π Σ Φ Ψ Ω

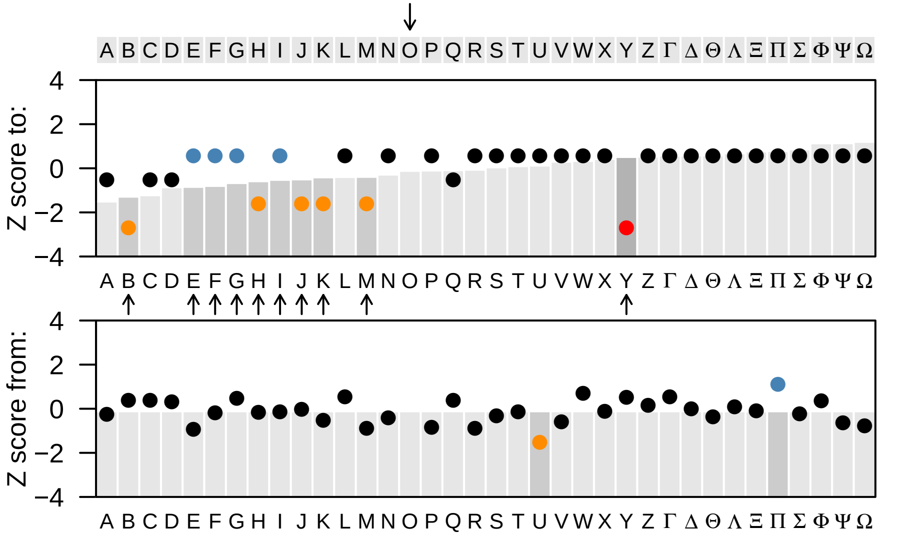

A B C D E F G H I J K L M N O P Q R S T U V W X Y Z Γ Δ Θ Λ Ξ Π Σ Φ Ψ Ω
Z score to:
Z score from:
4
2
0
−2
−4

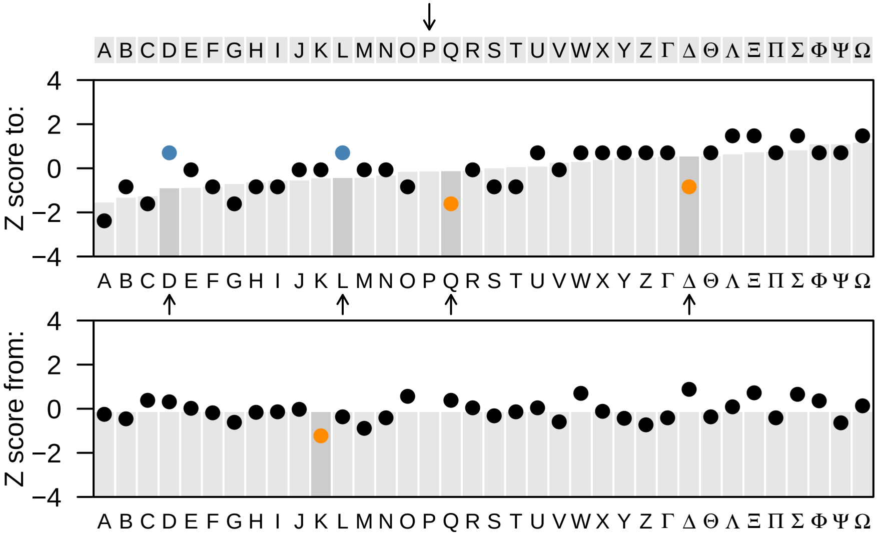
A B C D E F G H I J K L M N O P Q R S T U V W X Y Z Γ Δ Θ Λ Ξ Π Σ Φ Ψ Ω
Z score to:
4
2
0
−2
−4
A B C D E F G H I J K L M N O P Q R S T U V W X Y Z Γ Δ Θ Λ Ξ Π Σ Φ Ψ Ω
Z score from:
4
2
0
−2
−4
A B C D E F G H I J K L M N O P Q R S T U V W X Y Z Γ Δ Θ Λ Ξ Π Σ Φ Ψ Ω

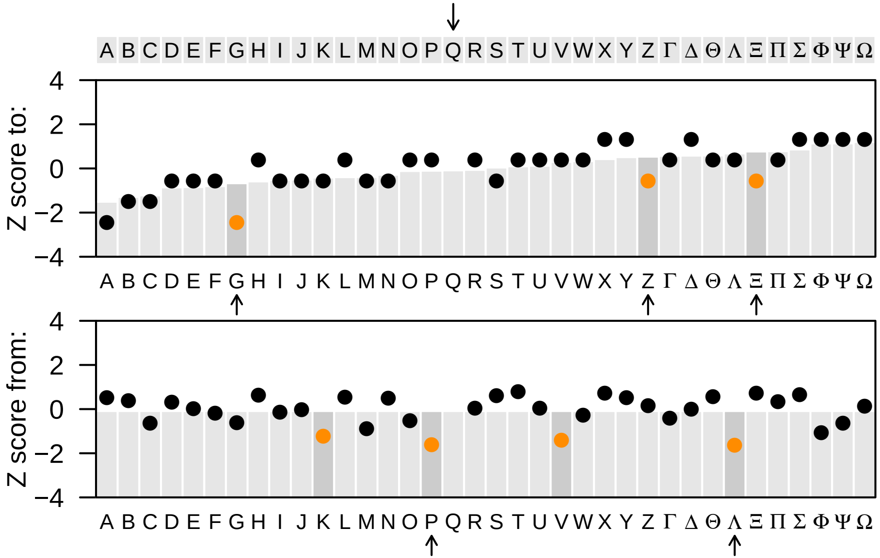

A B C D E F G H I J K L M N O P Q R S T U V W X Y Z Γ Δ Θ Λ Ξ Π Σ Φ Ψ Ω
Z score to:
4
2
0
−2
−4
Z score from:

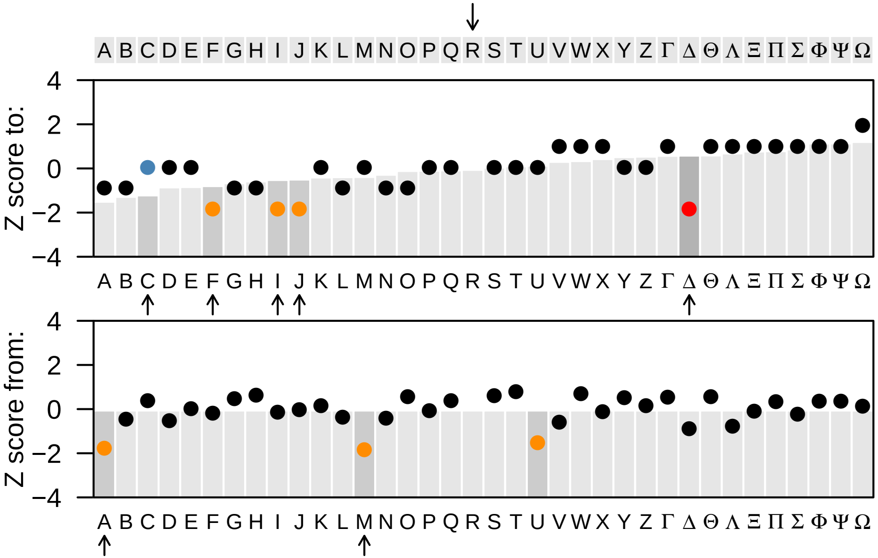

A B C D E F G H I J K L M N O P Q R S T U V W X Y Z Γ Δ Θ Λ Ξ Π Σ Φ Ψ Ω
Z score to:
4
2
0
−2
−4
A B C D E F G H I J K L M N O P Q R S T U V W X Y Z Γ Δ Θ Λ Ξ Π Σ Φ Ψ Ω
Z score from:
4
2
0
−2
−4
A B C D E F G H I J K L M N O P Q R S T U V W X Y Z Γ Δ Θ Λ Ξ Π Σ Φ Ψ Ω

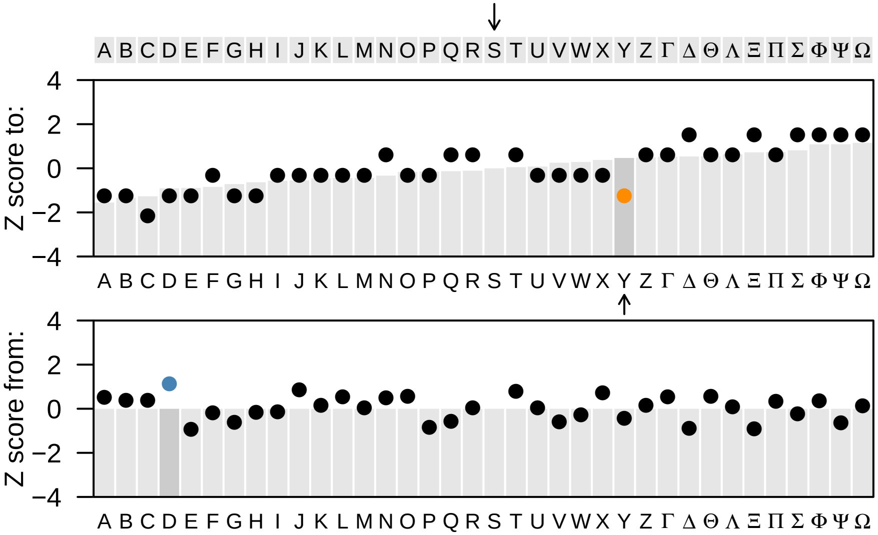

A B C D E F G H I J K L M N O P Q R S T U V W X Y Z Γ Δ Θ Λ Ξ Π Σ Φ Ψ Ω
Z score to:
4
2
0
−2
−4
Z score from:

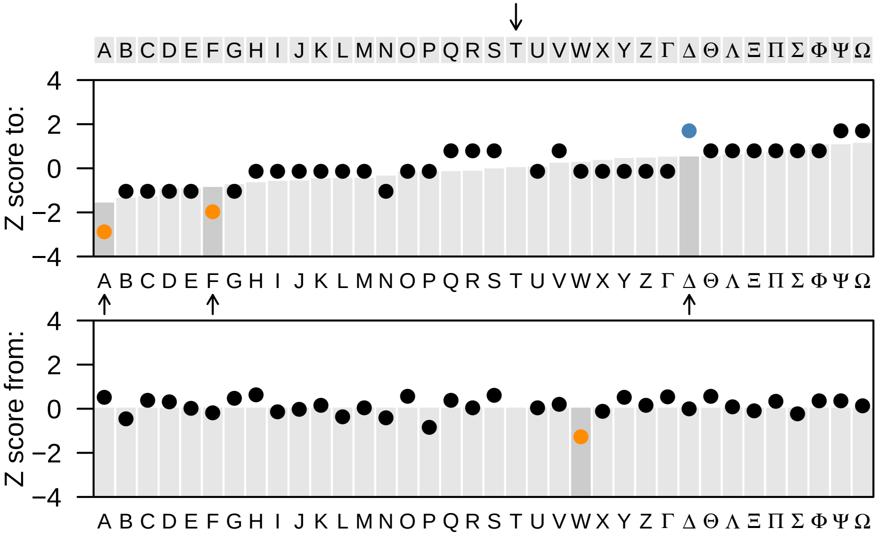

A B C D E F G H I J K L M N O P Q R S T U V W X Y Z Γ Δ Θ Λ Ξ Π Σ Φ Ψ Ω
Z score to:
Z score from:
4
2
0
−2
−4

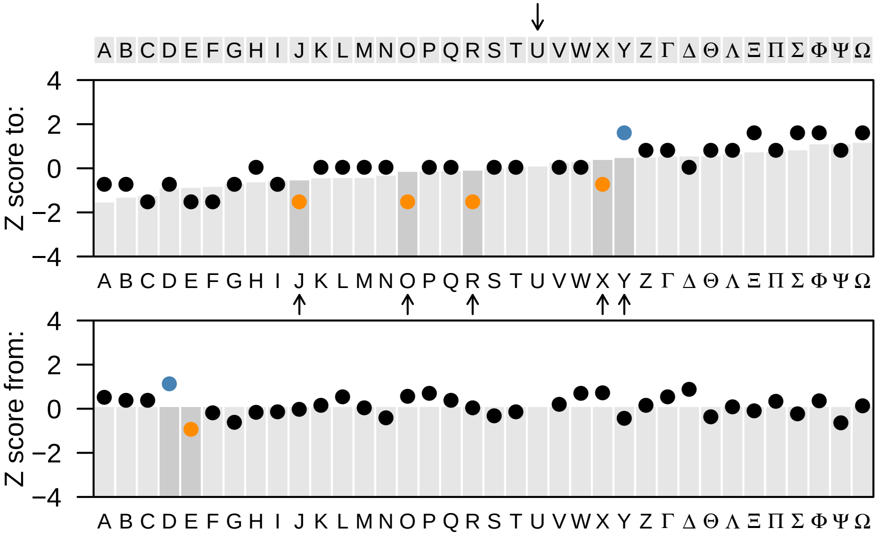
A B C D E F G H I J K L M N O P Q R S T U V W X Y Z Γ Δ Θ Λ Ξ Π Σ Φ Ψ Ω
Z score to:
Z score from:
4
2
0
−2
−4

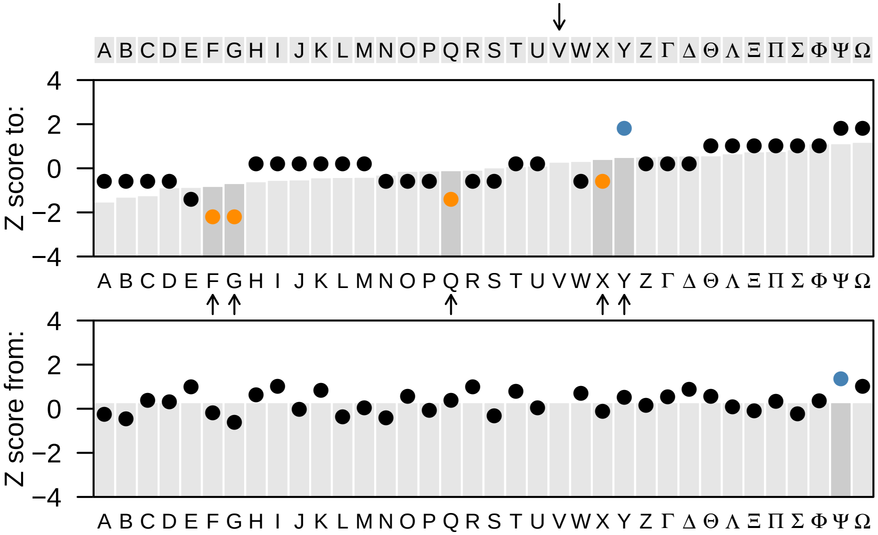

A B C D E F G H I J K L M N O P Q R S T U V W X Y Z Γ Δ Θ Λ Ξ Π Σ Φ Ψ Ω
Z score to:
Z score from:
4
2
0
−2
−4

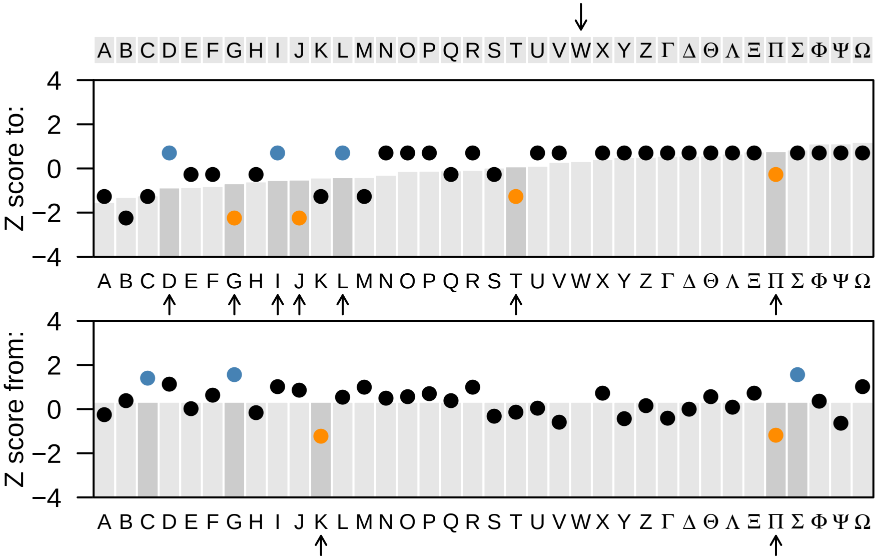

A B C D E F G H I J K L M N O P Q R S T U V W X Y Z Γ Δ Θ Λ Ξ Π Σ Φ Ψ Ω
Z score to:
Z score from:
4
2
0
−2
−4

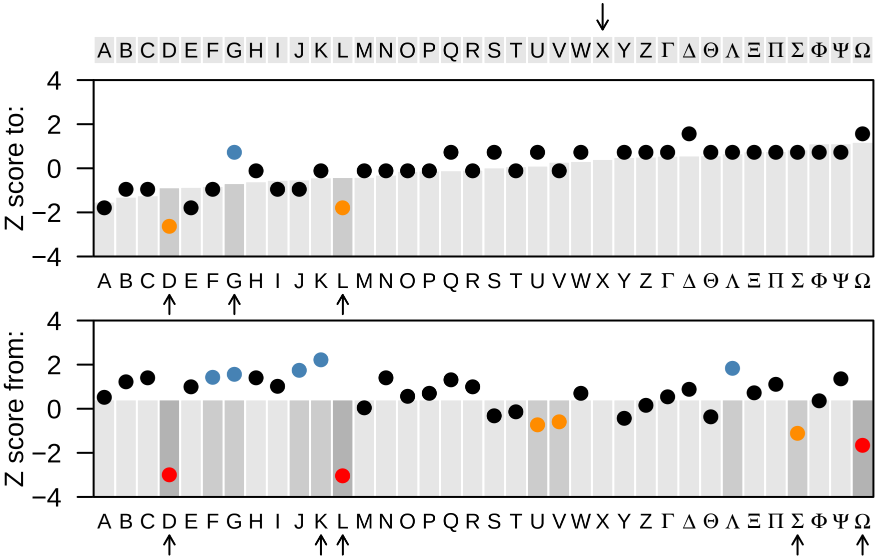

A B C D E F G H I J K L M N O P Q R S T U V W X Y Z Γ Δ Θ Λ Ξ Π Σ Φ Ψ Ω
Z score to:
4
2
0
−2
−4
Z score from:

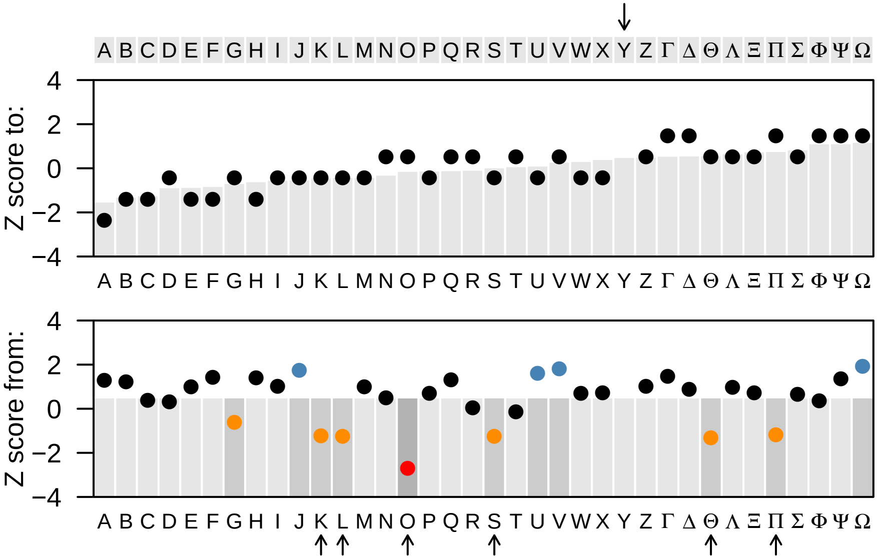
A B C D E F G H I J K L M N O P Q R S T U V W X Y Z Γ Δ Θ Λ Ξ Π Σ Φ Ψ Ω
Z score to:
4
2
0
−2
−4
Z score from:

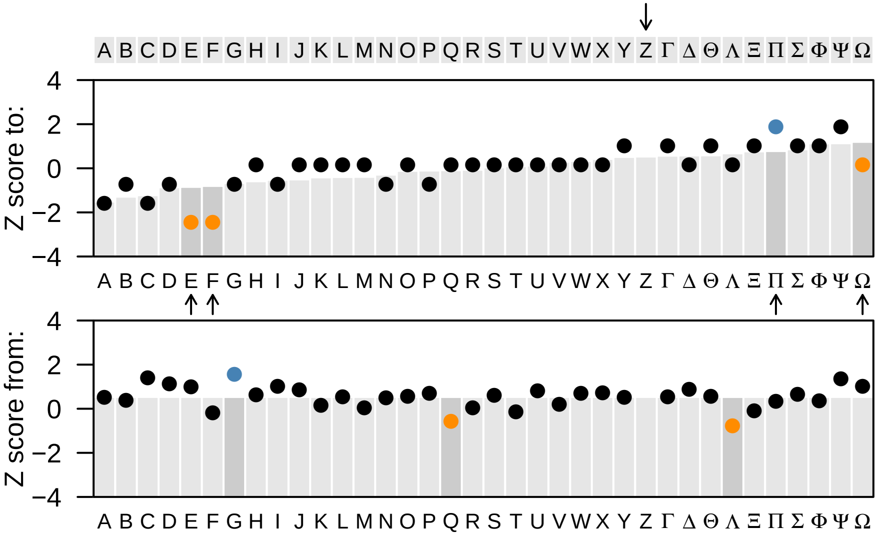

A B C D E F G H I J K L M N O P Q R S T U V W X Y Z Γ Δ Θ Λ Ξ Π Σ Φ Ψ Ω
Z score to:
Z score from:
4
2
0
−2
−4

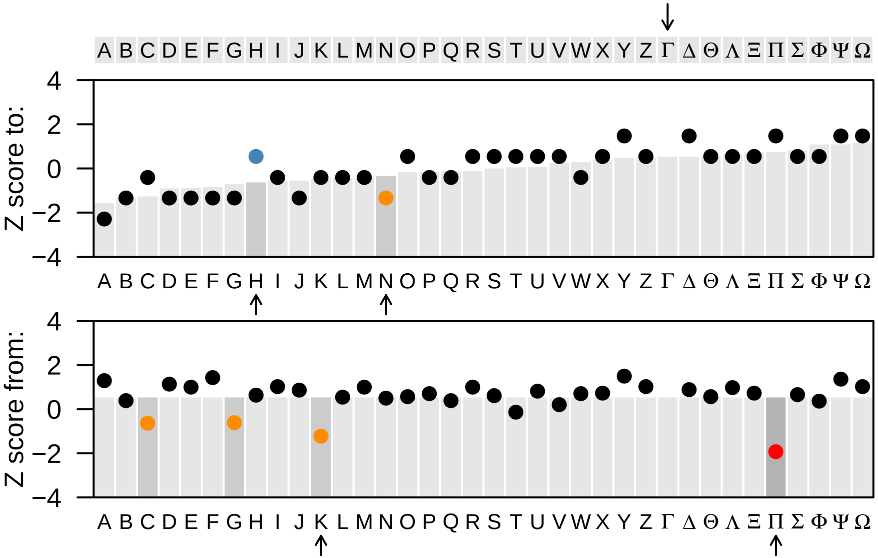

A B C D E F G H I J K L M N O P Q R S T U V W X Y Z Γ Δ Θ Λ Ξ Π Σ Φ Ψ Ω
Z score to:
Z score from:
4
2
0
−2
−4

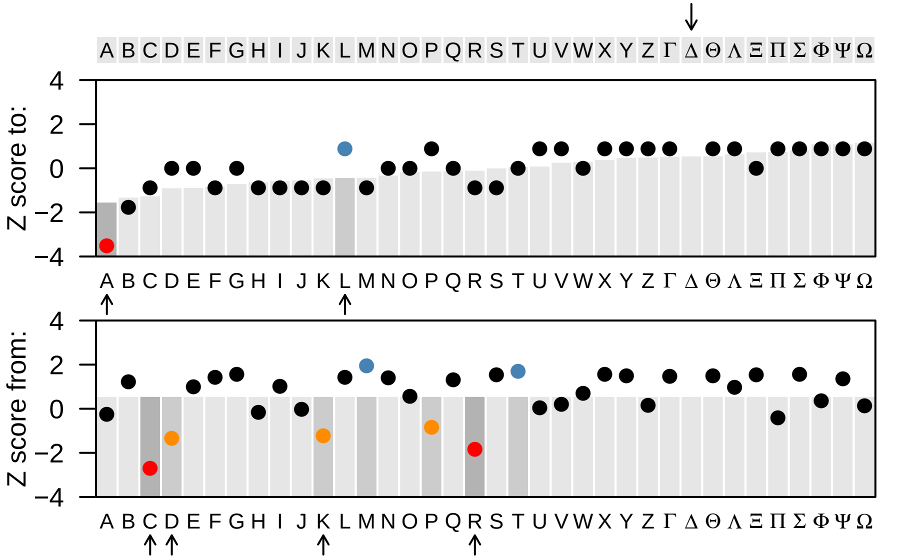
A B C D E F G H I J K L M N O P Q R S T U V W X Y Z Γ Δ Θ Λ Ξ Π Σ Φ Ψ Ω
Z score to:
4
2
0
−2
−4
Z score from:

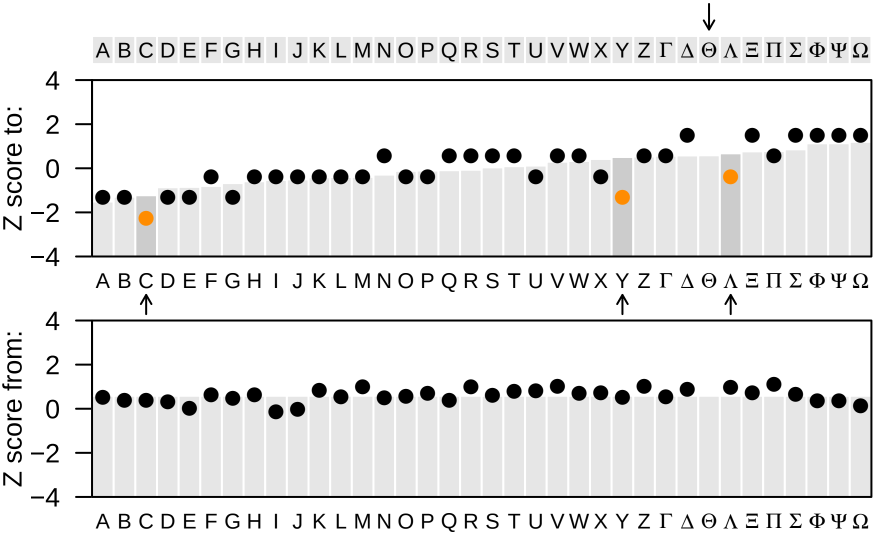
A B C D E F G H I J K L M N O P Q R S T U V W X Y Z Γ Δ Θ Λ Ξ Π Σ Φ Ψ Ω
Z score to:
4
2
0
−2
−4
A B C D E F G H I J K L M N O P Q R S T U V W X Y Z Γ Δ Θ Λ Ξ Π Σ Φ Ψ Ω
Z score from:
4
2
0
−2
−4
A B C D E F G H I J K L M N O P Q R S T U V W X Y Z Γ Δ Θ Λ Ξ Π Σ Φ Ψ Ω

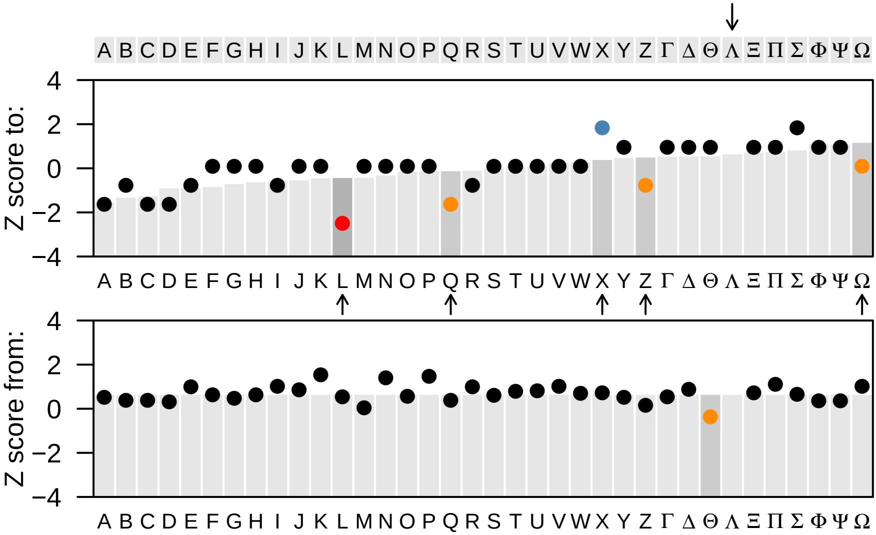

A B C D E F G H I J K L M N O P Q R S T U V W X Y Z Γ Δ Θ Λ Ξ Π Σ Φ Ψ Ω
Z score to:
Z score from:
4
2
0
−2
−4

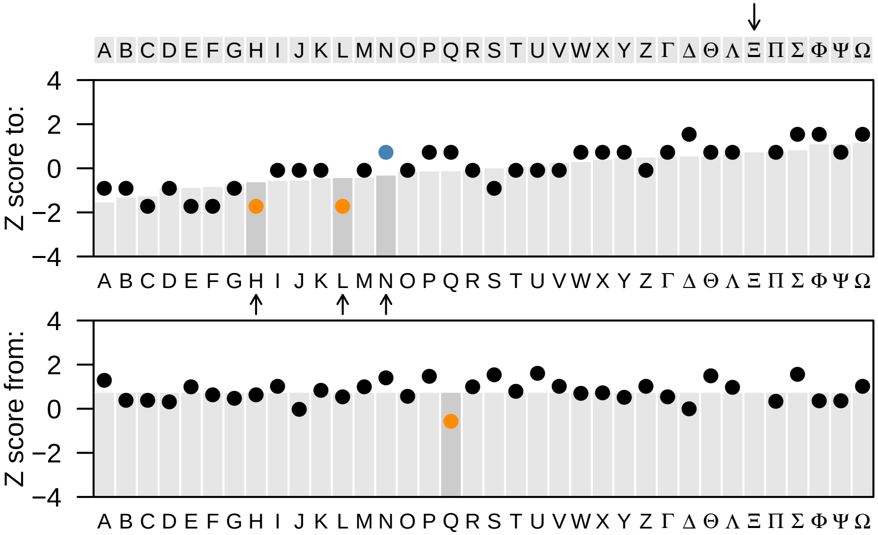

A B C D E F G H I J K L M N O P Q R S T U V W X Y Z Γ Δ Θ Λ Ξ Π Σ Φ Ψ Ω
Z score to:
Z score from:
4
2
0
−2
−4

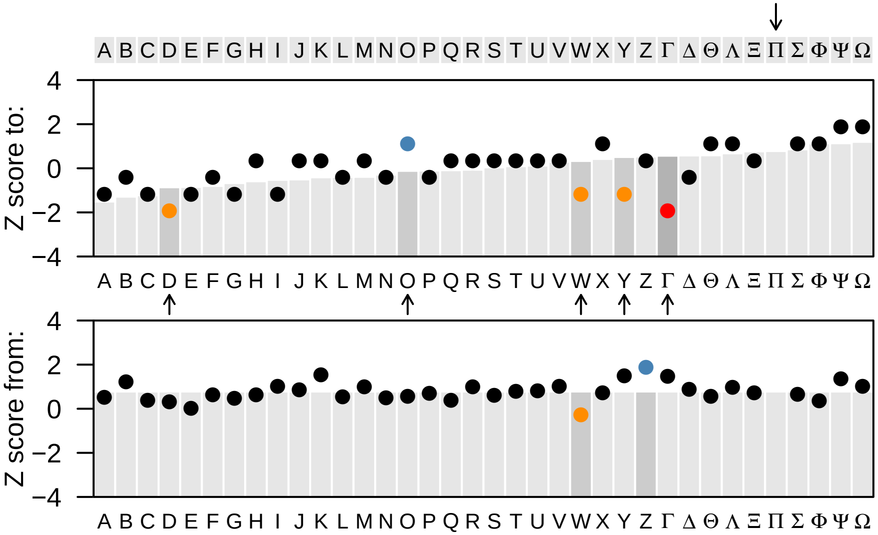
Z score to:
Z score from:
4
2
0
−2
−4
A B C D E F G H I J K L M N O P Q R S T U V W X Y Z Γ Δ Θ Λ Ξ Π Σ Φ Ψ Ω

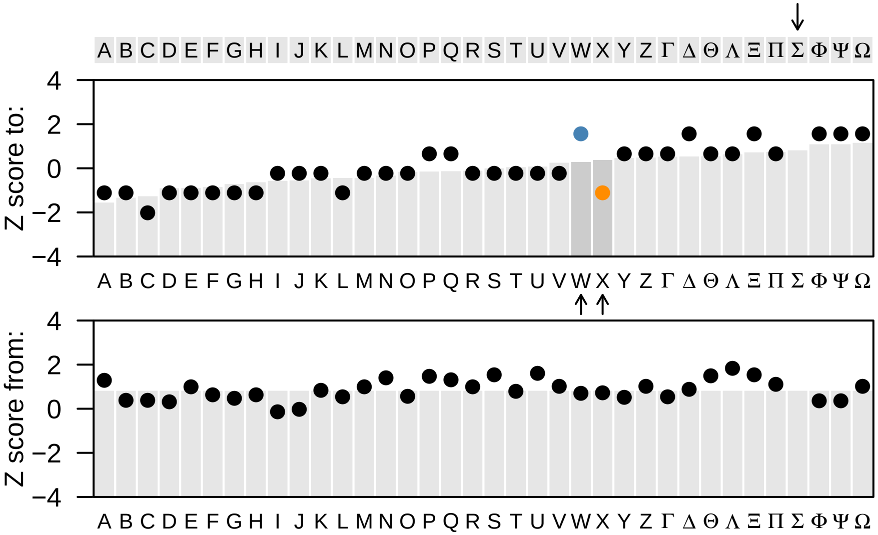

A B C D E F G H I J K L M N O P Q R S T U V W X Y Z Γ Δ Θ Λ Ξ Π Σ Φ Ψ Ω
Z score to:
4
2
0
−2
−4
Z score from:

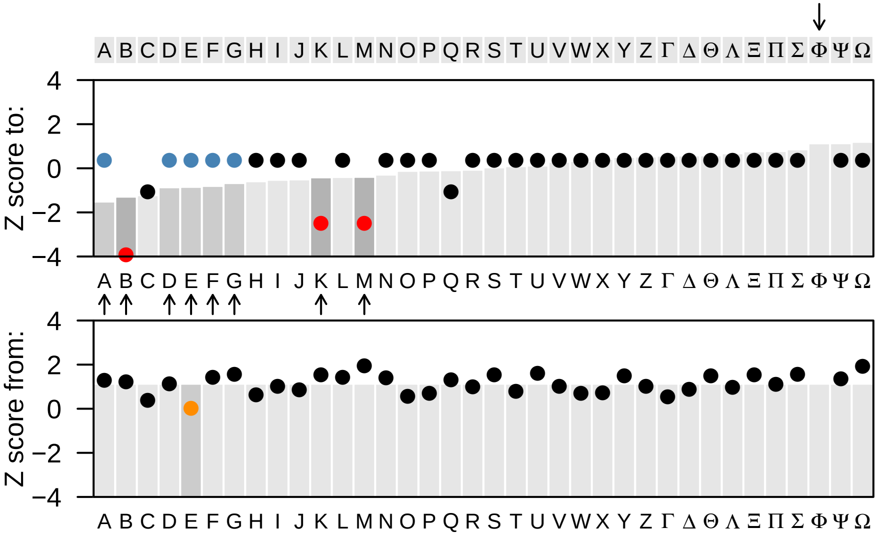

A B C D E F G H I J K L M N O P Q R S T U V W X Y Z Γ Δ Θ Λ Ξ Π Σ Φ Ψ Ω
Z score to:
4
2
0
−2
−4
Z score from:

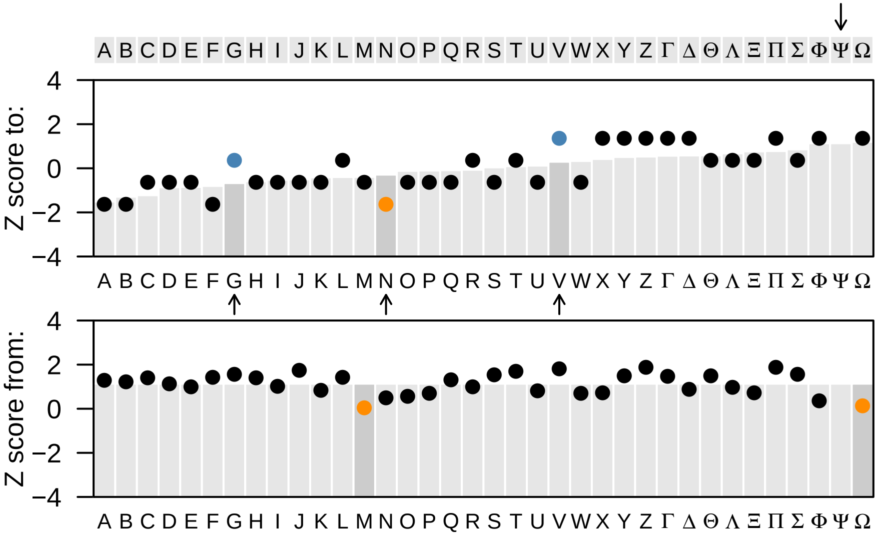

A B C D E F G H I J K L M N O P Q R S T U V W X Y Z Γ Δ Θ Λ Ξ Π Σ Φ Ψ Ω
Z score to:
Z score from:
4
2
0
−2
−4

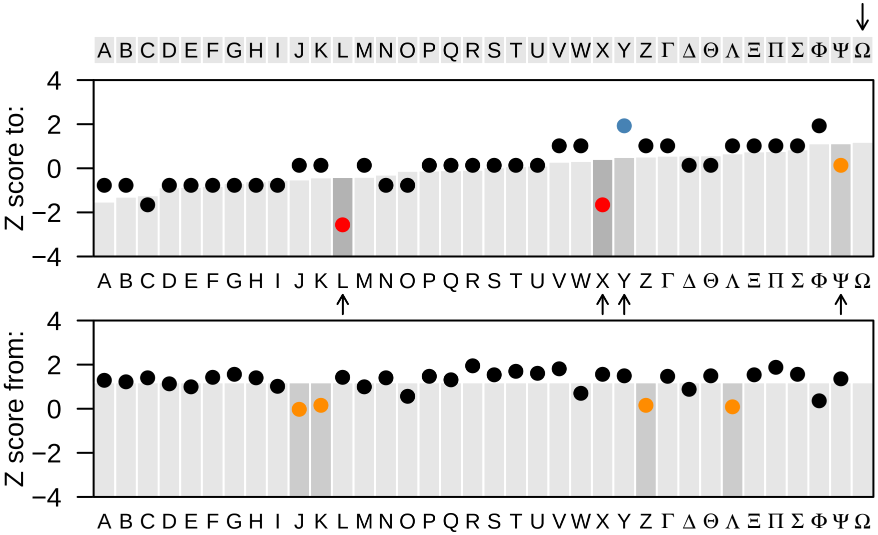

A B C D E F G H I J K L M N O P Q R S T U V W X Y Z Γ Δ Θ Λ Ξ Π Σ Φ Ψ Ω
Z score to:
Z score from:
4
2
0
−2
−4
