# Supplementary material for: Using peer review to distribute group work marks equitably between medical students
Source: BMC Med Educ. 2017 Sep 20;17:172. doi: 10.1186/s12909-017-0987-z (PMC5607620; doi:10.1186/s12909-017-0987-z)
Supplement: Supplementary file 3 — CHP 2014 Module Feedback Form. Form used to solicit feedback on the scoring system. (PDF 13 kb) [file 12909_2017_987_MOESM3_ESM.pdf]

YONG LOO LIN SCHOOL OF MEDICINE
UNDERGRADUATE STUDENT FEEDBACK
CHP MODULE FEEDBACK

Module Name : **MD4150 CHP**

Year of Study: 1 2 3 [4] 5

CHP Module Feedback *(circle accordingly)*

1 CHP provided an opportunity to learn about teamwork.

Excellent	Very Good	Good	Fair	Poor
5	4	3	2	1

2 CHP provided an opportunity to apply theory into practice in research.

Excellent	Very Good	Good	Fair	Poor
5	4	3	2	1

3 CHP enhanced my interest in public health.

Excellent	Very Good	Good	Fair	Poor
5	4	3	2	1

4 CHP enhanced my interest in research.

Excellent	Very Good	Good	Fair	Poor
5	4	3	2	1

5 CHP helped me better understand a health need or problem in the community.

Excellent	Very Good	Good	Fair	Poor
5	4	3	2	1

6 CHP was a good learning experience.

Excellent	Very Good	Good	Fair	Poor
5	4	3	2	1

7 I rate the CHP module as:

Excellent	Very Good	Good	Fair	Poor
5	4	3	2	1

In both past and present CHPs, 70% of the CHP mark is from Group Work scores and 30% of the CHP mark is from Individual Test scores. In past CHPs, Group Work scores were the sum of Group Presentation, Group Report and Peer Review scores. For present CHPs, Group Work scores are based on the sum of Group Presentation and Group Report scores but the Peer Review score is used to allocate a proportion of summed score which may be higher or lower than the summed score. The algorithm used will give a higher summed score if the student is assessed by peers to have contributed significantly to their group's CHP and a lower summed score if the student is assessed by peers to have under-contributed to their group's CHP. The algorithm used will also control for inter-group and intra-group variations so that the score will be fair across the whole class.

8 Which CHP grading system do you think is <u>fairer</u>?

Past / Present

9 Which CHP grading system do you <u>prefer</u>?

Past / Present

Turn to next page

10 Any further comments/suggestions for the CHP:
